# Emerging Trends of Multidrug-Resistant (MDR) and Extensively Drug-Resistant (XDR) *Salmonella* Typhi in a Tertiary Care Hospital of Lahore, Pakistan

**DOI:** 10.3390/microorganisms9122484

**Published:** 2021-11-30

**Authors:** Muhammad Zakir, Maryam Khan, Muhammad Ihtisham Umar, Ghulam Murtaza, Muhammad Ashraf, Saba Shamim

**Affiliations:** 1 Institute of Molecular Biology and Biotechnology, The University of Lahore, Defense Road Campus, Lahore 54000, Pakistan; Muhammad.zakir06@gmail.com (M.Z.); maryamkkhan246@gmail.com (M.K.); ashrafbot@yahoo.com (M.A.); 2 Department of Pharmacy, COMSATS University Islamabad, Lahore Campus, Lahore 54000, Pakistan; ihtishamumar@cuilahore.edu.pk (M.I.U.); gmdogar@cuilahore.edu.pk (G.M.)

**Keywords:** *Salmonella* Typhi, multidrug-resistant, extensively drug-resistant, typhoid fever, *Salmonella enterica* serovar Typhimurium, Lahore

## Abstract

*Salmonella* Typhi is a Gram-negative pathogen that causes typhoid fever in humans. The use of antibiotics to treat typhoid has considerably mitigated its fatality risk, but rising multidrug-resistant (MDR) and extensively drug-resistant (XDR) resistance in Pakistan threatens effective treatment. This study determined the prevalence of MDR and XDR *S*. Typhi at a local hospital in Lahore. Blood samples (*n* = 3000) were obtained and processed for bacterial identification. Antibiotic susceptibility test was performed using VITEK^®^ 2 Compound 30 System. Statistical data analysis was performed using a Mann–Whitney U and Kruskal–Wallis H test, respectively. The results revealed 600 positive cultures, of which the majority were found to be XDR *S*. Typhi (46.1%) and MDR *S*. Typhi (24.5%) strains. The disease burden of resistant *Salmonella* strains was greater in males (60.67%) than females (39.33%), with the most affected age group being 0–10 years old (70.4 %). In both the outpatient department (OPD) and general ward, the prevalence of XDR *S*. Typhi cases was found to be alarmingly high (48.24%), followed by MDR *S*. Typhi (25.04 %). The results of the statistical analysis demonstrated that the incidence of resistance in MDR and XDR *S*. Typhi strains was not affected by the age as well as the gender of patients (*p* > 0.05). The occurrence of resistant strains against four tested antibiotics (azithromycin, ciprofloxacin, imipenem, and meropenem) was found to be similar in different wards and among hospitalized and OPD patients (*p* > 0.05). Maximum resistance was observed against chloramphenicol and ampicillin in the OPD and pediatric ward. Piperacillin/Tazobactam was observed to be the most effective antibiotic, followed by co-amoxiclav (*p* < 0.001). This study is effective in validating the existence of MDR and XDR *S*. Typhi in Lahore, where stringent methods should be applied for controlling its spread.

## 1. Introduction

Typhoid fever is caused by the Gram-negative pathogen known as *Salmonella enterica* serovar Typhi, which is responsible for contributing greatly to the disease burden of the world, with more than 14 million cases of typhoid and paratyphoid fever in 2017, respectively, which caused more than 130,000 fatalities with approximately 70% of the fatality burden occurring in South Asia [1].

Chloramphenicol was the first antibiotic to be used for treating typhoid in the 1940s [2,3,4], but the subsequent resistance against it prompted the administration of other antibiotics such as co-trimoxazole in the 1970s. However, reports in the 1980s described the resistance of *S*. Typhi strains against all previously used antibiotics [5]. Due to this resistance against chloramphenicol, other antibiotics such as ampicillin and trimethoprim-sulfamethoxazole became treatment of choice for typhoid, despite their lesser efficacious potential than other drugs prescribed before them. However, it was not long before the emergence of resistance against these two drugs was also reported around the world, with increasing fatality in cases also being synonymous with the resistant strains. This resulted in turning towards fluoroquinolones such as ciprofloxacin, which were then used for treating enteric and typhoid fever [6]. Unfortunately, emerging multidrug-resistant (MDR) and extensively drug-resistant (XDR) *S*. Typhi strains have wreaked havoc on the efficacy of many antibiotics, with only a few options remaining for the treatment of benign and severe cases of typhoid fever. On these grounds, MDR *S*. Typhi strains are generally regarded to be resistant against at least one out of three or more than three categorically differentiated antimicrobials, such as ampicillin, sulfonamides (trimethoprim-sulfamethoxazole), and chloramphenicol, whereas XDR are those which are observed to be resistant to all but one or two antimicrobials, demonstrating resistance against several types of antibiotics such as chloramphenicol, ampicillin, sulfonamides, fluoroquinolones (ciprofloxacin), and third-generation cephalosporins (ceftazidime, cefuroxime, and ceftriaxone), leaving out few options such as piperacillin/tazobactam, azithromycin, and carbapenems as efficient treatment options [7,8]. Moreover, since their use, resistance to fluoroquinolones was observed not so long ago, with most of the cases being reported to originate and disseminate from South Asia. Consequently, hope has been resorted to cephalosporins and azithromycin for potential treatment where clinical evidence suggests their use to be currently efficacious, though cephalosporin-resistant *S*. Typhi has also been reported in the world, contributing greatly to the disease burden of typhoid and enteric fever in South Asia as well as other parts of the world where MDR and XDR *S*. Typhi strains are dominantly found to cause disease [9].

In Pakistan, the incidence of typhoid caused by MDR and XDR *S*. Typhi has been on the increase, fueling fears of antibiotic treatment failure [10]. In 2016–2017, more than 800 cases of XDR typhoid were reported in Hyderabad alone, marking the whole region endemic with respect to typhoid [11,12]. The year 2016 also saw the emergence of the first report of XDR *S*. Typhi in Karachi. Ever since the first outbreak in Sindh, more than 17,000 cases of XDR typhoid have been reported [13]. Although many studies in Pakistan have been previously limited to Sindh, there have been reports of emerging cases nationwide [14,15] and elsewhere due to provincial and international travel [16]. Moreover, a rise in typhoid cases with similar clinical manifestations to COVID has been observed during the pandemic, with more than 20,000 cases being diagnosed in Pakistan in the month of June 2020 [17].

This research study was performed to determine the prevalence of MDR and XDR *S.* Typhi from a tertiary care hospital in Lahore in the province of Punjab, Pakistan. The patterns of antibiotic sensitivity were also observed to study the resistance behavior of XDR and MDR *S*. Typhi isolates, respectively. 

## 2. Materials and Methods

### 2.1. Sampling and Data Collection

Blood cultures of 3000 subjects were obtained from the microbiology laboratory of Ittefaq Hospital, Lahore, over ten months (May 2020–February 2021), which were then processed by the VersaTREK™ Automated Microbial Detection System (ThermoFisher Scientific, Waltham, MA, USA) for the evidence of microbial growth, gas formation, and hemolysis. Each of the blood culture vials was monitored for seven consecutive days after its incubation at 37 °C. After seven days, a blood culture vial with no evidence of visible microbial growth was sub-cultured before ruling it to be negative for the patient. For the positive blood culture vials, the samples were cultured onto basic, enriched, and selective media for the growth of the suspected pathogen [18]. Patients of positive samples were also asked about their medical history and background in a brief proforma. 

### 2.2. Antibiotic Susceptibility Test

The antibiotic susceptibility of samples was identified and evaluated by the VITEK^®^ 2 Compact 30 system (Biomerieux, Marcy-l’Étoile, France). The isolates were tested against a panel of 13 antibiotics, namely amoxicillin, ampicillin, azithromycin, chloramphenicol, ciprofloxacin, ceftazidime, cefuroxime, ceftriaxone, imipenem, meropenem, cefoperazone-sulbactam, trimethoprim-sulphamethoxazole, and piperacillin/tazobactam using the GN ID (Gram-negative identification) cassette. The card was placed at room temperature for 15 min prior to opening the package liner. In the next step, 3 mL of sterile saline solution was placed aseptically in a clear polystyrene test tube with the help of sterile swabs. The homogeneous suspension was prepared by placing pure colonies from the culture plates into the saline solution test tubes. The suspension was adjusted using McFarland standard (0.5–0.6) by using a calibrated Densichek meter. The prepared suspension was then placed into the cassette, while the GN ID straw was placed into the machine simultaneously. The data of the patients were entered, and the results were observed and compiled over the course of 8–10 h. Strains demonstrating susceptibility to all antibiotics were termed *Salmonella* species (non-typhoidal *Salmonella*), whereas *Salmonella* Typhi and Paratyphi A strains were characterized as being resistant to ciprofloxacin only. Strains resistant to at least three different categories of antimicrobials were considered MDR *S*. Typhi, while strains resistant to all but two or more antimicrobials were termed XDR *S*. Typhi, respectively [4,8,19].

### 2.3. Statistical Analysis

The statistical analyses were performed by using The Statistical Package for the Social Sciences (SPSS) version 26 (SPSS Inc., Chicago, IL, USA). The variance in the incidence of antibiotic resistance with age, gender, patient status (hospitalized or non-hospitalized), hospital ward, and the microbial pathogen was statistically analyzed by a Mann–Whitney U test and a Kruskal–Wallis H test. The null hypothesis (the incidence of antibiotic resistance was independent of the gender, age, patient hospitalization status, ward, and the microbial pathogen) was rejected at the alpha (*p*) value < 0.05. Moreover, the patient demographic details, as well as the mean percentages for age and gender, were also calculated.

## 3. Results

### 3.1. Distribution of MDR and XDR Salmonella Strains According to Gender and Age

**Figure 1:**
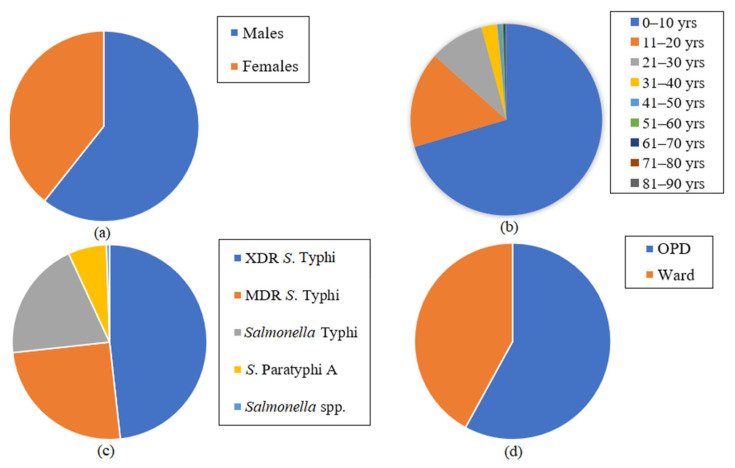
The distribution of typhoid fever cases according to (**a**) gender, (**b**) age, (**c**) cases in Outpatient Department (OPD) and hospital ward, and (**d**) *Salmonella* strains (XDR *S*. Typhi, MDR *S*. Typhi, *S*. Typhi, *S*. Paratyphi A, and *Salmonella* spp.) in both OPD and ward sections of the hospital.

From a total of 3000 samples, 600 cultures were observed to be positive for *Salmonella*, of which the majority were XDR *S*. Typhi (*n* = 276, 46.1%), followed by MDR *S*. Typhi (*n* = 147, 24.5%), *S*. Typhi (*n* = 128, 21.3%), *S*. Paratyphi A (*n* = 38, 6.3%), and *Salmonella* spp. (non-typhoidal *Salmonella*) (*n* = 11, 1.8%), respectively. Out of MDR *S*. Typhi strains, two isolates were observed to demonstrate additional resistance patterns due to which they were labeled MDR 4, while the rest were labeled MDR 1, respectively. However, they were categorized as one category (MDR *S*. Typhi) for the rest of the study. The disease burden of resistant MDR and XDR *S*. Typhi strains was found to be greater in males (*n* = 364, 60.67%) as compared to females (*n* = 236, 39.33%) (Figure 1a). Moreover, among the nine categorized age groups, most of the cases were reported from 0–10 years (*n* = 419, 69.83%), followed by 11–20 years (*n* = 96, 16.42%), 21–30 years (*n* = 55, 9.16%), 31–40 years (*n* = 16, 2.6%), 41–50 years (*n* = 4, 0.66%), 51–60 and 61–70 years (*n* = 2, 0.5%), and 81–90 years (*n* = 1, 0.33%), respectively (Figure 1b).

### 3.2. Distribution of MDR and XDR Salmonella Strains in OPD and Ward Cases

In comparison to the patients receiving hospital care in its wards, the Outpatient Department (OPD) section saw more cases of typhoid fever being treated (57.98%), while the hospital wards treated fewer cases (42.02%) (Figure 1c). Moreover, of both OPD and ward cases, the prevalence of XDR *S*. Typhi cases was found to be alarmingly high (48.24%), followed by MDR *S*. Typhi cases (25.04%), *S.* Typhi cases (19.83 %), S. Paratyphi A (6.39%), and *Salmonella* spp. (non-typhoidal *Salmonella*) strains (0.5%), in both OPD and ward cases (Figure 1d). 

### 3.3. Association of Related Factors with Antibiotic Resistance of XDR and MDR S. Typhi Strains

The statistical analysis was performed by applying a Mann–Whitney U test and a Kruskal–Wallis H test, and the alpha (*p*) values for the analyzed factors are presented in Table 1 below. The relation of each factor was considered statistically significant at *p* < 0.05.

### 3.4. Resistance of Different Salmonella Strains against Co-Amoxiclav, Ampicillin, Chloramphenicol, Ceftazidime, Cefixime, and Ciprofloxacin

**Figure 2:**
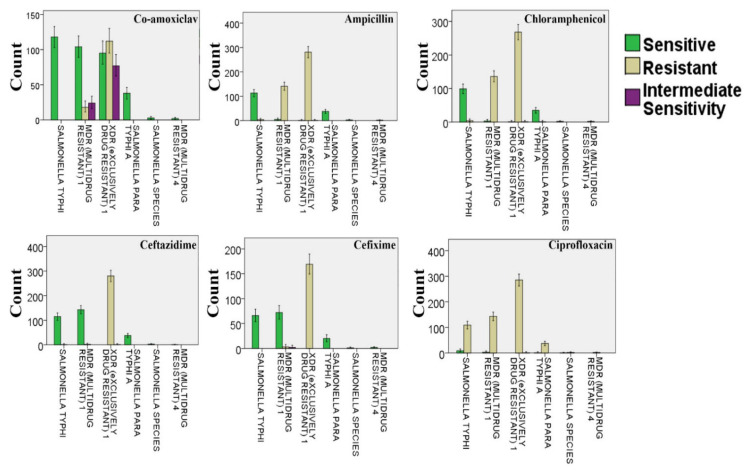
Resistance of different *Salmonella* strains against co-amoxiclav, ampicillin, chloramphenicol, ceftazidime, cefixime, and ciprofloxacin. The bars present the number of cases with resistant, sensitive, and intermediately sensitive strains against the mentioned antibiotics, whereas the error bars present a 95% confidence interval.

Widespread resistance to ciprofloxacin and chloramphenicol (*n* => 250 isolates) was observed, whereas XDR *S*. Typhi strains were also found to be resistant to ampicillin and cefixime. MDR *S*. Typhi strains were found to be resistant to ampicillin and chloramphenicol but sensitive to cefixime, ceftazidime, and co-amoxiclav (Figure 2). 

### 3.5. Resistance of Different Salmonella Strains against Ceftriaxone, Cefoperazone/Sulbactam, Co-Trimoxazole, and Piperacillin/Tazobactam

**Figure 3:**
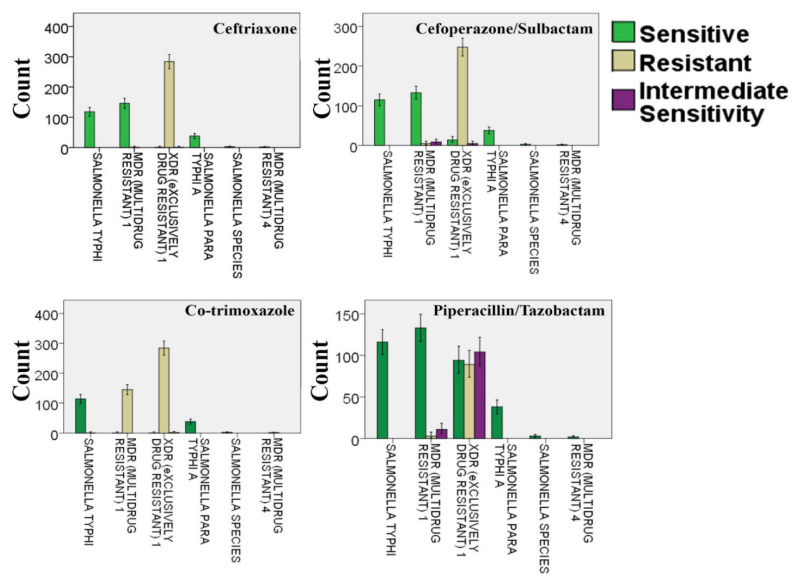
Resistance of *Salmonella* strains against ceftriaxone, cefoperazone/sulbactam, co-trimoxazole, and piperacillin/tazobactam. The bars present the number of cases with resistant, sensitive, and intermediately sensitive strains against the mentioned antibiotics whereas the error bars present a 95% confidence interval.

The resistance of XDR *S*. Typhi strains was observed in the case of all antibiotics, whereas in the case of MDR *S*. Typhi strains, resistance was observed against co-trimoxazole. *S.* Typhi was found to be sensitive against all antibiotics (Figure 3). 

### 3.6. Resistance of MDR and XDR S. Typhi against Co-Amoxiclav, Ampicillin, and Chloramphenicol in OPD and Ward Patients

**Figure 4:**
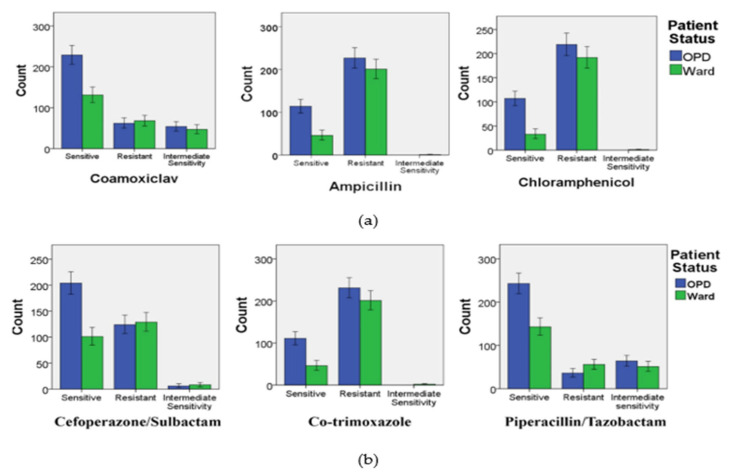
The occurrence of resistant *S*. Typhi strains against (**a**) co-amoxiclav, ampicillin, and chloramphenicol and (**b**) cefoperazone, co-trimoxazole, and piperacillin/tazobactam in OPD and ward patients.

In the case of co-amoxiclav, the number of resistant isolates was more in the ward cases than the OPD cases. The difference between the number of sensitive isolates in the OPD and ward patients was found to be statistically significant (*p* < 0.05). In the case of ampicillin, OPD cases saw a greater number of resistant strains. A similar pattern was observed in the case of chloramphenicol in both OPD and ward cases (Figure 4a). The error bars indicate a 95% confidence interval. 

### 3.7. Resistance of MDR and XDR S. Typhi against Cefoperazone/Sulbactam, Co-Trimoxazole, and Piperacillin/Tazobactam in OPD and Ward Patients

The most resistance was observed in OPD cases against co-trimoxazole, as compared to ward cases (*p* < 0.05). In the case of cefoperazone/sulbactam, most of the bacterial strains that were isolated from the outpatients were found to be sensitive, whereas the resistant strains and their subsequent cases in the hospital ward were in greater number than in OPD. Likewise, a similar sensitivity pattern was observed in the case of piperacillin/tazobactam (Figure 4b). The error bars indicate a 95% confidence interval.

### 3.8. Resistance of MDR and XDR S. Typhi against Co-Amoxiclav in Various Hospital Wards

The most sensitivity and resistance to co-amoxiclav was found in the OPD and Peads (pediatric) ward (Band Bagum Ward) of the hospital (*p* < 0.05), respectively (Figure S1). The error bars indicate a 95% confidence interval. No significant cases of sensitivity and resistance were observed in other wards of the hospital, respectively. 

### 3.9. Resistance Pattern of MDR and XDR S. Typhi against Ampicillin in Various Hospital Wards

In the OPD section of the hospital, resistance to the strains was greatly observed, whereas sensitivity was less against ampicillin (*p* < 0.05). A similar trend of resistance was found in the pediatric ward of the hospital (Figure S2), but the sensitivity ratio was much less when compared to that of the OPD section. The error bars of the graph indicate a 95% confidence interval.

### 3.10. Resistance Pattern of MDR and XDR S. Typhi against Chloramphenicol in Various Hospital Wards

Results were similar to ampicillin, resistance in the strains was greatly observed in the OPD and pediatric ward of the hospital against chloramphenicol (*p* < 0.05). The error bars of the graph indicate a 95% confidence interval (Figure S3). 

### 3.11. Resistance Pattern of MDR and XDR S. Typhi against Ceftazidime in Various Hospital Wards

An increasing trend of sensitivity against ceftazidime was found in the cases being treated in the OPD section of the hospital, whereas the trend was inverse in the case of the pediatric ward. The sensitivity and resistance in both sections of the hospital were found to be statistically significant (*p* < 0.05). The error bars of the graph indicate a 95% confidence interval (Figure S4).

### 3.12. Resistance Pattern of MDR and XDR S. Typhi against Ceftriaxone in Various Hospital Wards

An increasing trend of sensitivity against ceftriaxone was found in the cases being treated in the OPD section of the hospital, whereas the trend was opposite in the case of pediatric ward. The sensitivity and resistance in both sections of the hospital were found to be statistically significant (*p* < 0.05). The error bars of the graph indicate a 95% confidence interval (Figure S5).

### 3.13. Resistance Pattern of MDR and XDR S. Typhi against Cefoperazone/Sulbactam in Various Hospital Wards

In the OPD section, increased sensitivity against cefoperazone/sulbactam was observed, which was statistically significant (*p* < 0.05). In both sections of the hospital, intermediate sensitivity to the antibiotic was also reported. The error bars of the graph indicate a 95% confidence interval (Figure S6).

### 3.14. Resistance Pattern of MDR and XDR S. Typhi against Piperacillin/Tazobactam in Various Hospital Wards

Increased sensitivity to piperacillin/tazobactam was observed in OPD, whereas intermediate sensitivity was found to be greater than the resistance pattern of the strains reported in OPD cases (*p* < 0.05). In the pediatric ward of the hospital, sensitivity was increased but the resistance pattern was greater than the intermediate sensitivity cases. The error bars of the graph indicate a 95% confidence interval (Figure S7).

## 4. Discussion

The worldwide incidence of typhoid fever presents a grave challenge to public health and the economic infrastructure of many developing and developed countries. More than 15 million cases of typhoid and paratyphoid fever were reported globally in 2015, of which the greatest burden of disease was reported in poverty-stricken regions of Asia, Southeast Asia, and Sub-Saharan Africa [20]. As of recently, this number has been reported to increase drastically, with more than 20 million cases being reported every year globally. The global impact of typhoid fever is more pronounced due to the emergence of antimicrobial resistance, such as the prevalence of drug resistant *S*. Typhi in high-risk countries [21,22,23]. Moreover, the occurrence of MDR and XDR *S*. Typhi cases in Pakistan have also increased at an alarming rate, with an elevated risk of an infectious outbreak in the provinces of Punjab and Sindh [10].

This study was conducted to examine the prevalence of MDR and XDR *S.* Typhi at a tertiary care hospital in Lahore, Pakistan. The blood cultures obtained from patients were proceeded and were found to be positive for *S*. Typhi, which is a cause of increasing concern in the local population. Treating MDR and XDR *S*. Typhi has presented itself to be a great challenge for doctors and clinicians in Lahore and Pakistan on the whole, due to the poor socio-economic status of a typical patient of the country. To further complicate the issue, the characteristic treatment of MDR and XDR typhoid is deemed to be expensive. In Pakistan, many doctors, as well as patients, unfortunately favor the empirical route of treatment, where patients often seek the help of over-the-counter medicine or untrained medical professionals who do not have the required skill of handling infectious diseases. In this regard, children often belong to the high-risk group for various diseases including typhoid fever [24]. 

In this study, the incidence of resistance in XDR and MDR *S*. Typhi strains was not affected by the age or the gender of the patients (*p* > 0.05), and the trend of isolating resistant pathogens against all the tested antibiotics was found to be statistically the same in both genders and all age groups of the patients (Table 1). In this study, males (60.67%) were found to be more affected than females (39.33%) (Figure 1a). The age group most affected by the incidence of typhoid and paratyphoid fever cases in our study was observed to be 0–10 years (70.4%), followed by the age bracket of 11–20 years, respectively (Figure 1b). The present study finding coincides with the previously established fact that typhoid is the most common bacterial illness that afflicts children in Pakistan, with more than 1000 cases per 100,000 children in Karachi alone [25,26]. The results of our study were in agreement with the study findings of Saeed et al. [14], where most cases occurred in children less than 15 years of age. Moreover, another study also reported the dominance of typhoid cases in children [27]. The prominent pattern of infection in the male population has similarities in many studies investigating endemic breakouts in countries, with a greater number of culture-confirmed typhoid and paratyphoid fever cases occurring in males [28,29,30]. Yousafzai et al. [31] also reported males to be affected more than females in their study. This predisposition offers no biological explanation, but a logical one demonstrates the high number to be attributable to the relatively greater exposure to the outer environments where contamination rates can run high. Furthermore, the higher tendency of males to dine outdoors and eat commercially prepared food and a more casual outlook regarding illness may be the reason for a high number of infections in males [32]. 

For the treatment of typhoid and paratyphoid fever, the preferred treatment was the use of first-generation antibiotics (ampicillin, chloramphenicol, and co-trimoxazole) between the 1940s and the 1990s, respectively. Sadly, the incessant use of these antibiotics accelerated the rise of resistance to these drugs, firstly to chloramphenicol, then to ampicillin and eventually co-trimoxazole, giving rise to MDR *S*. Typhi strains. MDR typhoid emerged in Asia in the 1990s, where the establishment of resistant strains and their resistance mechanisms were acquired via horizontal gene transfer and integrons, plasmids, and transposons, which encode resistant genes [33]. The most common plasmids conferring resistance in *S*. Typhi strains at the time were reported to be the IncHI1 type [34]. Insight into the plasmid type suggest that it was first obtained by H58 type and other haplo-types of *S*. Typhi around the 1990s. With the emergence of MDR *S*. Typhi, the use of first-generation antibiotics became superannuated in many regions of the world [35].

In low- and middle-income countries, resistance against antibiotics such as macrolides, fluoroquinolones, and β-lactams has been acquired due to their misuse of over-the-counter prescriptions, which facilitates the transfer of resistance genes very easily. Similar happenings in countries such as Pakistan and India have positioned them among the top three countries with the largest consumption of antibiotics. Its plausible explanation was attributable to the over-prescription or inadequate prescription practices that favor many second- and third-generation antibiotics, where a large portion of typhoid cases in Pakistan receive a prescription without the confirmation of the pathogen through blood culture or lack of diagnostic facilities and for their administration in typhoid and paratyphoid fever along with many other medical conditions such as respiratory, GIT, genitourinary tract infections, and skin and tissue infections amidst the emergence of resistance against broad and narrow-spectrum antibiotics, respectively [36].

Our study observed more typhoid cases in the OPD section (57.98%) as compared to hospital wards (42.02%) (Figure 1c), respectively, where the prevalence of XDR *S*. Typhi strains was found to be alarmingly high (Figure 1d). Our findings revealed the trend of the occurrence of resistant *S.* Typhi strains against the tested antibiotics (except azithromycin, ciprofloxacin, imipenem, and meropenem), which was found to be significantly different in various wards of the hospital (*p* < 0.05). Moreover, the resistance pattern against the mentioned antibiotics was also found to be different in the patients who were admitted to the wards and OPD section of the hospital (*p* < 0.05). Like the age and gender of the patients, the occurrence of resistant bacteria against four tested antibiotics, i.e., azithromycin, ciprofloxacin, imipenem, and meropenem, was found to be similar in different wards of the hospital and the OPD section (*p* > 0.05). Although the resistant strains against all tested antibiotics were isolated from different wards, the maximum resistance was recorded against ampicillin and chloramphenicol in the OPD and pediatric ward of the hospital. Likewise, the incidence of resistance against ampicillin and chloramphenicol was greater in the OPD patients as compared to the hospitalized patients (Figure 2). The results of our study were in agreement with the study findings of Tewari et al. [18], where MDR and XDR strains of *S.* Typhi demonstrated resistance against first-generation antibiotics including ampicillin and chloramphenicol. Almost all XDR *S*. Typhi strains were found to be resistant to ceftriaxone, cefoperazone/sulbactam, co-trimoxazole, and piperacillin/tazobactam (Figure 3). Recently, two studies conducted in Pakistan elucidated the presence of MDR and XDR *S*. Typhi strains, which were resistant against several antibiotics such as co-trimoxazole, ampicillin, chloramphenicol, and ciprofloxacin [37,38]. 

Our study demonstrated a statistically significant difference in the resistance among *S.* Typhi strains against all tested antibiotics except azithromycin, imipenem, and meropenem. Most of the resistant isolates were found to be XDR (extensively drug-resistant) against all tested antibiotics. Furthermore, a majority of the strains were resistant to ciprofloxacin, for which the number of resistant strains was significantly higher than the sensitive strains (*p* < 0.001). Contrarily, piperacillin/Tazobactam was found to be the most effective antibiotic. Moreover, the number of XDR *S*. Typhi strains was also less than the total number of sensitive strains for piperacillin/tazobactam. Co-amoxiclav was the second most effective drug against all tested strains (except XDR *S*. Typhi) with a higher number of sensitive strains as compared to the resistant ones (*p* < 0.001) (Figure 4a,b). Resistance to fluoroquinolones is mediated through the changes in the conformation of DNA gyrase as well as topoisomerase IV, which are the main sites of fluoroquinolone action [39,40,41]. The results of the study were in agreement with the study findings of Fatima et al. [37], where major MDR and XDR S. Typhi strains were found to be sensitive to piperacillin/tazobactam. In a recent study, the emergence of XDR *S*. Typhi was reported in Lahore, which was likely due to the constant travel of individuals at the provincial level, bringing in the resistant strain from Sindh to Punjab, as well as the usage of ineffective or counterfeit antibiotics, encouraging transmission of the resistant strains at a national level. Moreover, XDR *S*. Typhi was reported to be resistant to several antibiotics such as ciprofloxacin, co-trimoxazole, ampicillin, ceftriaxone, and chloramphenicol, which was in congruence with the results of our study [42] (Figures S1–S7). The emergence of ciprofloxacin-resistant *S*. Typhi strains was also reported nationally as well as on a global level, respectively [43,44].

## 5. Conclusions

This study was conducted to determine the prevalence of MDR and XDR *Salmonella* Typhi at a tertiary care hospital in Lahore, Pakistan. The results revealed that the most resistance was observed in the case of ciprofloxacin and chloramphenicol against the majority of strains, whereas XDR *S*. Typhi strains were also found to be resistant against ampicillin and cefixime. MDR *S*. Typhi strains were found to be resistant to ampicillin and chloramphenicol but sensitive to cefixime, ceftazidime, and co-amoxiclav. Among these antibiotics, resistance against ciprofloxacin was found to be statistically significant (*p* < 0.05). Piperacillin/Tazobactam was found to be the most effective antibiotic. Furthermore, co-amoxiclav was the second most effective drug against all tested strains (except XDR *S*. Typhi) with a greater number of sensitive strains as compared to the resistant ones (*p* < 0.001). Although this study presents a comprehensive view of the occurrence of XDR and MDR *S*. Typhi, more data should be acquired from other hospitals so that it could span accurate information across the province and country. Moreover, increasing resistance to antibiotics demonstrates the swiftly deteriorating efficacy of antibiotics in the treatment of typhoid fever. Stringent methods should be adapted to curb the spread of the pathogen in the first place, and proper vaccination protocols for typhoid fever should be promptly regulated in places that are worst hit by resistant strains. Safety and hygiene practices should be taken into account as they primarily prevent pathogen dissemination.

## Figures and Tables

**Table 1:** The impact of gender, age, patient location (type of hospital ward), patient status (hospitalized or outdoor), and the type of *Salmonella* strains on the occurrence of antibiotic resistance was evaluated. The statistical analysis was performed by applying the Mann–Whitney U test (for patient gender and hospitalization status) and the Kruskal–Wallis H test (for age, patients’ ward, and pathogen).

Antibiotic	Factors				
	Age	Gender	Patient Location (Wards)	Patient Status (Ward/OPD)	Organism
Co-amoxiclav	0.08	0.886	**0.008**	**0.004**	**0.000**
Ampicillin	0.098	0.161	**0.001**	**0.000**	**0.000**
Azithromycin	0.751	0.105	0.508	0.486	0.467
Chloramphenicol	0.195	0.103	**0.000**	**0.000**	**0.000**
Ceftazidime	0.207	0.626	**0.000**	**0.000**	**0.000**
Cefixime	0.508	0.448	**0.003**	**0.001**	**0.000**
Ciprofloxacin	0.191	0.699	0.183	0.300	0.005
Ceftriaxone	0.200	0.545	**0.000**	**0.000**	**0.000**
Imipenem	0.665	0.254	0.249	0.228	0.325
Meropenem	0.532	0.214	0.154	0.240	0.487
Cefoperazone/Sulbactam	0.543	0.739	**0.000**	**0.000**	**0.000**
Co-trimoxazole	0.186	0.262	**0.001**	**0.000**	**0.000**
Piperacillin/Tazobactam	0.203	0.612	**0.001**	**0.000**	**0.000**

The alpha (*p*) values for the analyzed factors are presented, with each factor considered statistically significant at *p* < 0.05. The significant values are presented in bold in the table.

## Data Availability

All the data have been added to this article.

## Supplementary Materials

The following are available online at https://www.mdpi.com/article/10.3390/microorganisms9122484/s1, Figure S1: Occurrence of the resistant *Salmonella* strains against co-amoxiclav in different wards of the hospital. Figure S2: Occurrence of the resistant *Salmonella* strains against ampicillin in different wards of the hospital. Figure S3: Occurrence of the resistant *Salmonella* strains against chloramphenicol in different wards of the hospital. Figure S4: Occurrence of the resistant *Salmonella* strains against ceftazidime in different wards of the hospital. Figure S5: Occurrence of the resistant *Salmonella* strains against ceftriaxone in different wards of the hospital. Figure S6: Occurrence of the resistant *Salmonella* strains against cefoperazone/sulbactam in different wards of the hospital. Figure S7: Occurrence of the resistant *Salmonella* strains against piperacillin/tazobactam in different wards of the hospital.
