# Supplementary material for: Emerging Trends of Multidrug-Resistant (MDR) and Extensively Drug-Resistant (XDR) Salmonella Typhi in a Tertiary Care Hospital of Lahore, Pakistan
Source: Microorganisms. 2021 Nov 30;9(12):2484. doi: 10.3390/microorganisms9122484 (PMC8703728; doi:10.3390/microorganisms9122484)
Supplement: Supplementary file 1 [file microorganisms-09-02484-s001.zip › microorganisms-1403699-supplementary.pdf]

Supplementary Materials

Supplementary figures:

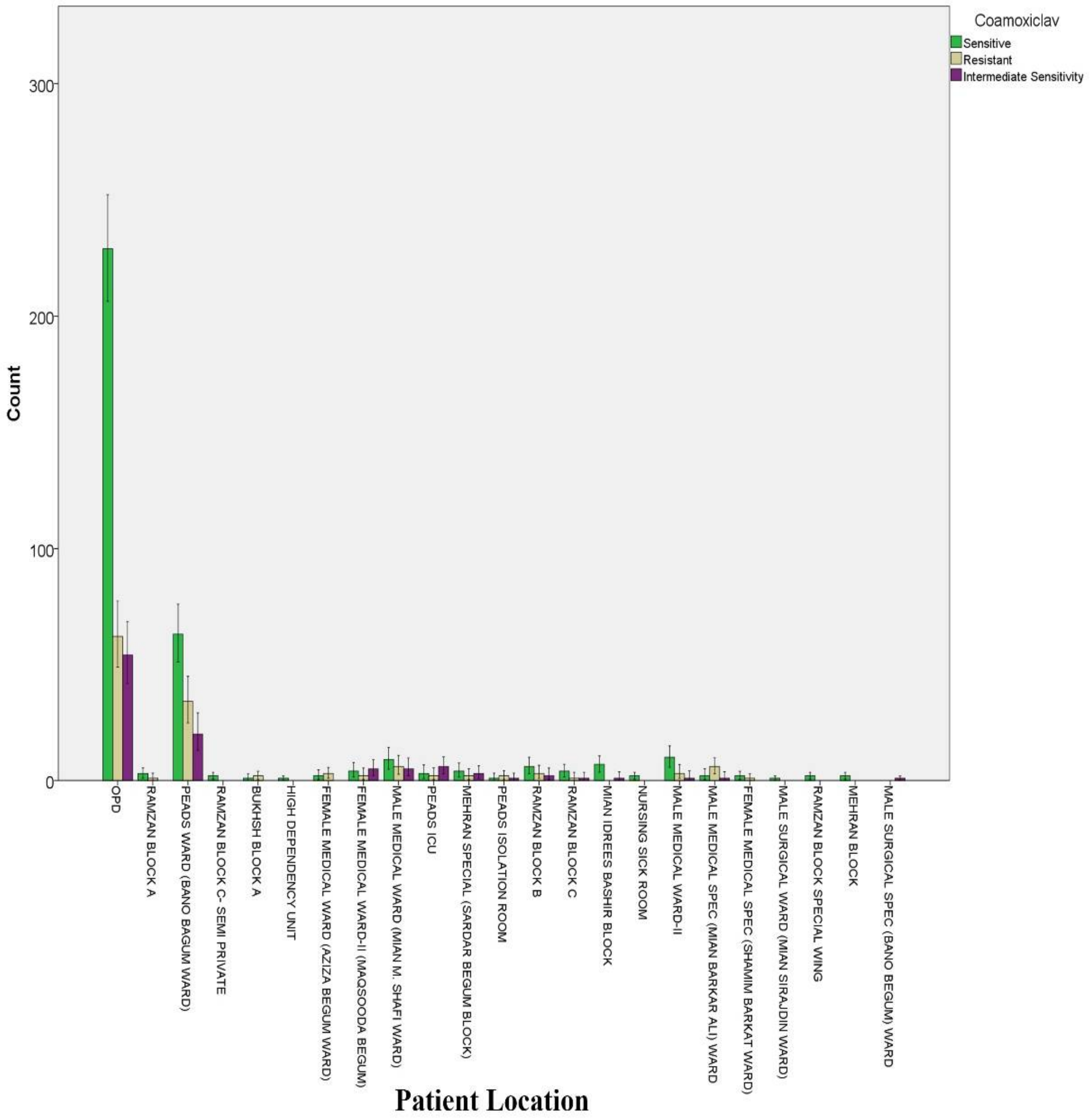

Figure S1. Occurrence of the resistant *Salmonella* strains against co-amoxiclav in different wards of the hospital.

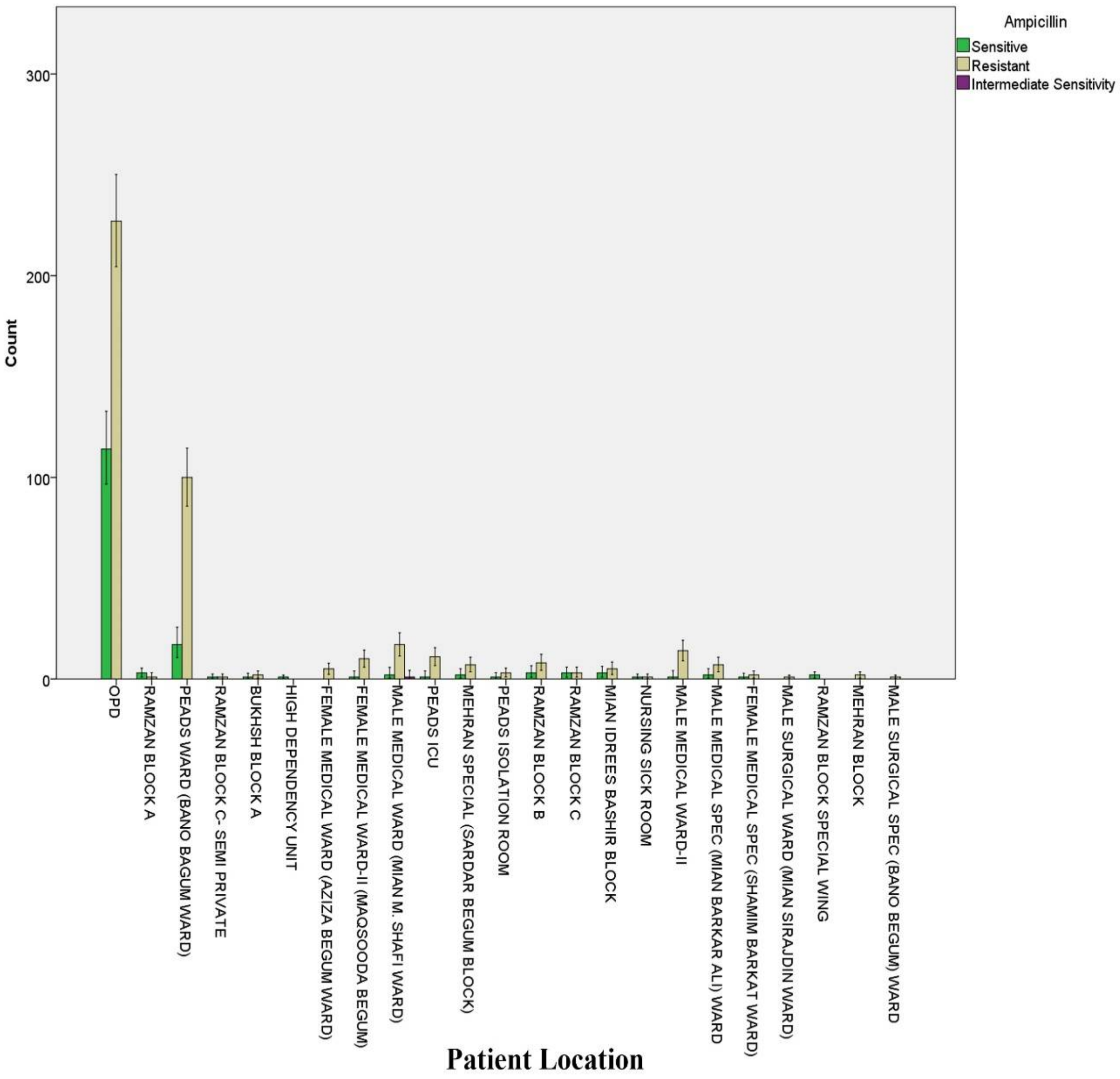

Figure S2. Occurrence of the resistant *Salmonella* strains against ampicillin in different wards of the hospital.

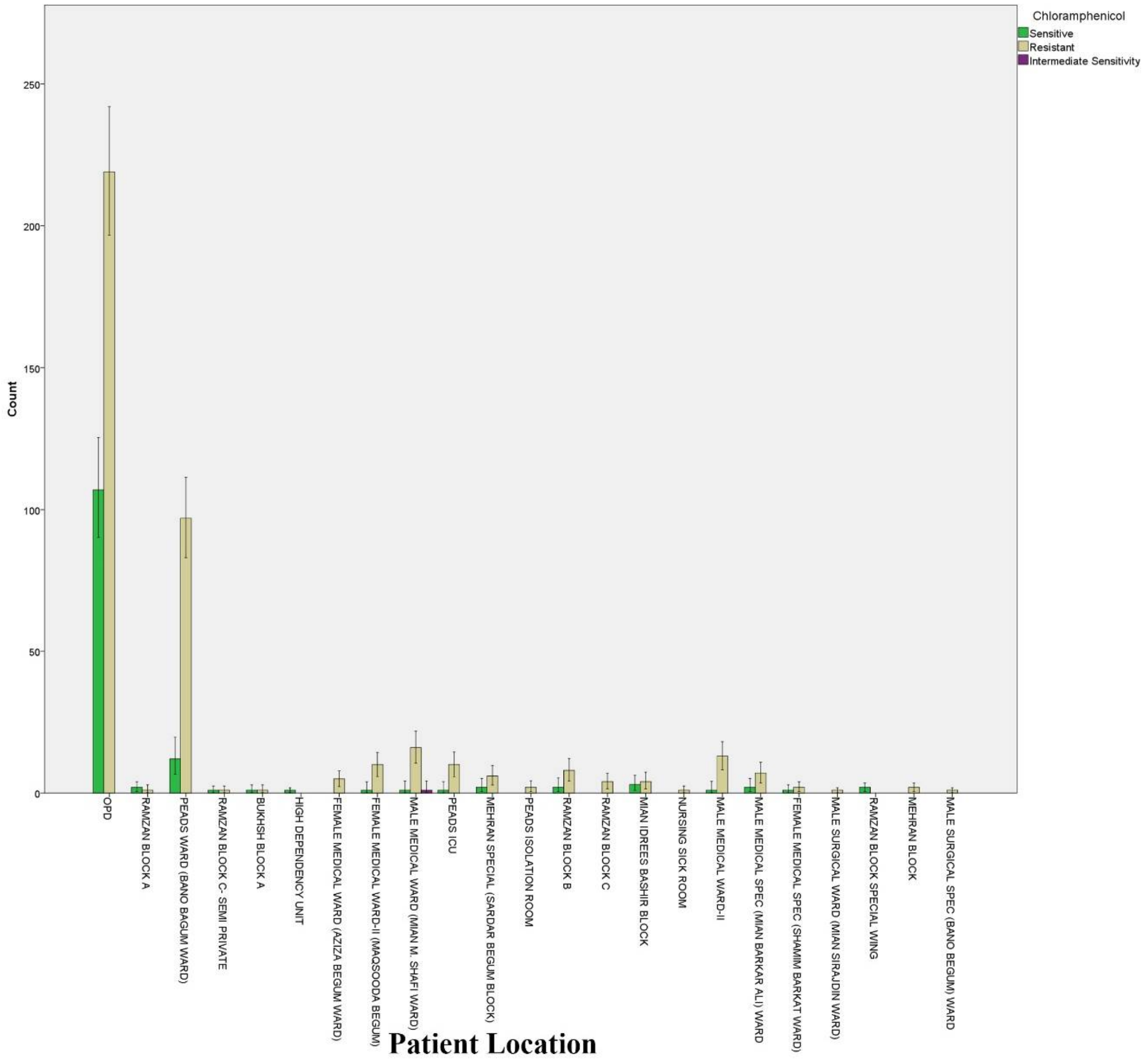

Figure S3. Occurrence of the resistant *Salmonella* strains against chloramphenicol in different wards of the hospital.

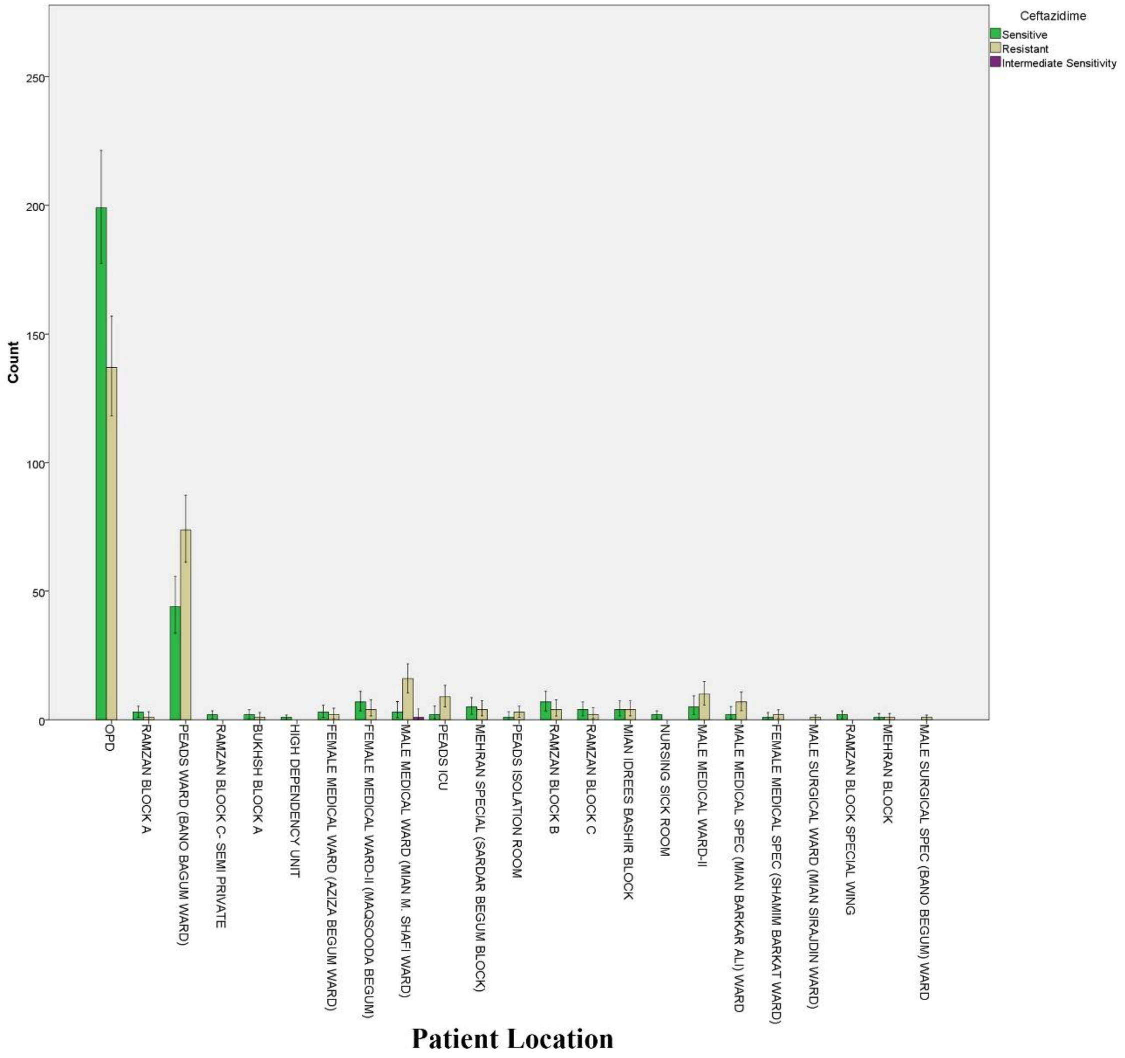

Figure S4. Occurrence of the resistant *Salmonella* strains against ceftazidime in different wards of the hospital

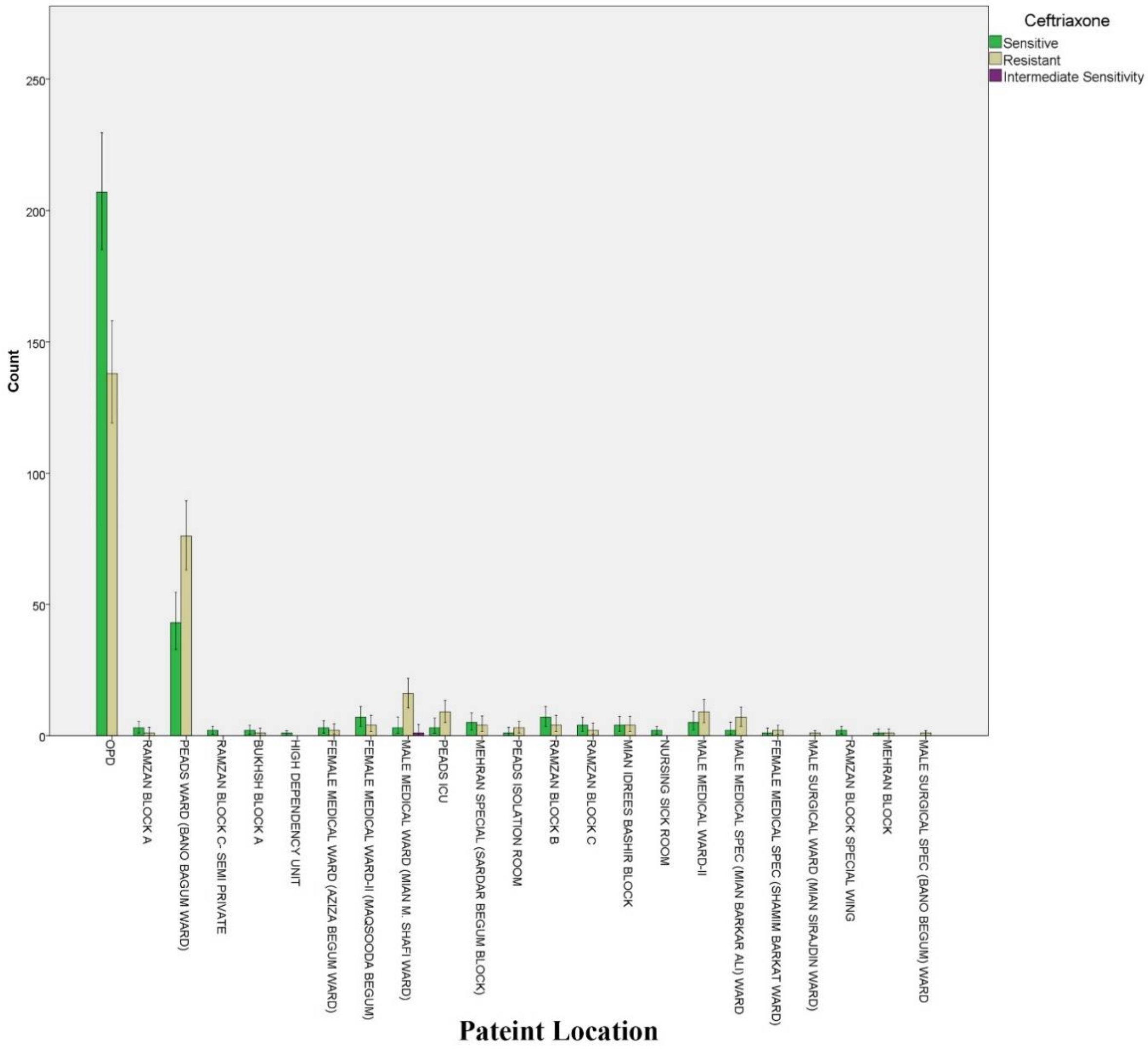

Figure S5. Occurrence of the resistant *Salmonella* strains against ceftriaxone in different wards of the hospital.

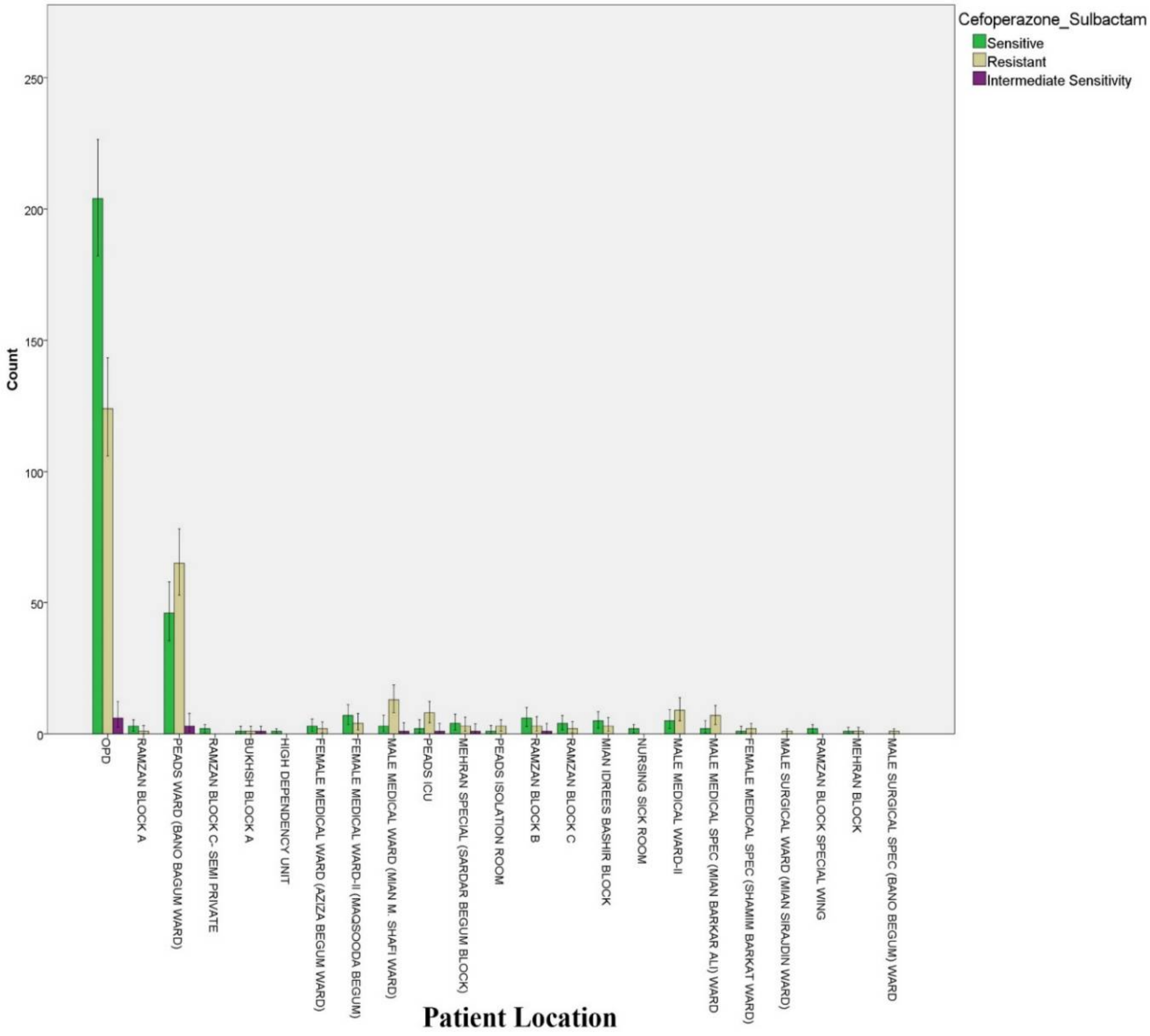

Figure S6. Occurrence of the resistant *Salmonella* strains against cefoperazone/sulbactam in different wards of the hospital.

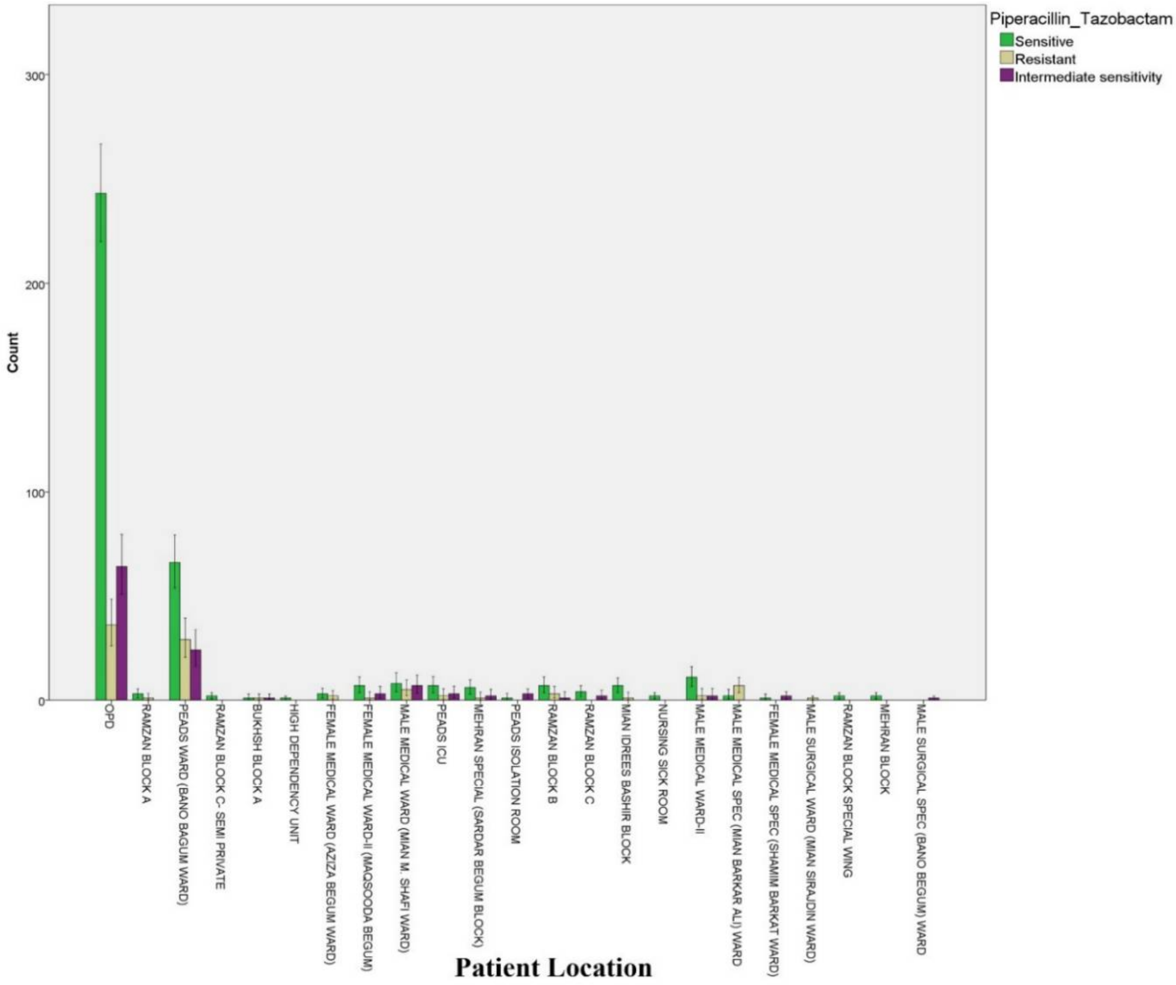

Figure S7. Occurrence of the resistant *Salmonella* strains against piperacillin/tazobactam in different wards of the hospital.
